# La COVID-19 et les priorités de recherche sur le vieillissement

**DOI:** 10.1017/S0714980820000343

**Published:** 2020-09-01

**Authors:** R. Jane Rylett, Flamine Alary, Joanne Goldberg, Susan Rogers, Patricia Versteegh

**Affiliations:** 1 Institut de recherche Robarts, Université de Western Ontario, London, Ontario; 2 Centre de recherche de l’Institut universitaire de gériatrie de Montréal, Université de Montréal, Québec

**Keywords:** aging, Canadian Institutes of Health Research, Institute of Aging, research prioritization, older adults, Covid-19, vieillissement, Instituts de recherche en santé du Canada, Institut du vieillissement, priorisation de la recherche, adultes plus âgés, Covid-19

## Abstract

Cet article présente les domaines prioritaires de recherche sur les impacts de la pandémie de COVID-19 chez les personnes âgées telles qu’ils ont été identifiés par l’Institut du vieillissement des IRSC (IV-IRSC). Le processus utilisé par l’IV-IRSC a comporté plusieurs phases itératives qui ont permis d’identifier trois secteurs prioritaires parmi les besoins de la recherche relative à la COVID-19, et quatre axes thématiques transversaux. Les secteurs de recherche prioritaires sont : 1) la réponse des personnes âgées à la maladie, à la vaccination et aux traitements, 2) la santé mentale et l’isolement, et 3) les milieux de soins soutenants. Les quatre thèmes transversaux sont : a) l’Équité, la diversité et l’inclusion (EDI), b) les considérations éthiques et morales, c) les pratiques fondées sur les données probantes, et d) les technologies numériques de la santé. Les priorités décrites dans cet article guideront les réponses de l’IV-IRSC aux besoins de la recherche sur la COVID-19.

## Abstract (EN)

This article describes priority areas for research on the impact of the Covid-19 pandemic on older adults that have been identified by the CIHR Institute of Aging (CIHR-IA). The process used by CIHR-IA consists of several iterative phases and thus far has resulted in identification of three key areas for Covid-19 research needs and four cross-cutting thematic areas. The key research priority areas are as follows: response of older adults to disease, vaccination, and therapeutics; mental health and isolation; and supportive care environments. The four cross-cutting themes are equity, diversity, and inclusion (EDI); ethical/moral considerations; evidence-informed practices; and digital health technologies. The priorities outlined in this article will inform CIHR-IA’s responses to Covid-19 research needs.

## Contexte

Le 11 mars 2020, l’Organisation mondiale de la Santé a annoncé que la COVID-19 avait atteint le statut de pandémie^1^. Peu de temps après, les activités de recherche à travers le Canada ont été drastiquement modifiées : les laboratoires et les installations de recherche ont fermé, et la plupart des chercheurs et des stagiaires ont adapté leurs recherches afin de pouvoir travailler, dans la mesure du possible, à partir de leur domicile. Simultanément à ces changements, il devint évident que les personnes âgées subissaient de manière disproportionnée les impacts négatifs de la COVID-19.

La mission de l’Institut du vieillissement des Instituts de recherche en santé du Canada (IV-IRSC) est d’appuyer la recherche, de promouvoir le vieillissement en santé et de se pencher sur les causes, la prévention, le dépistage, le diagnostic, le traitement, les systèmes de soutien et les soins palliatifs pour la prise en charge des problèmes de santé complexes qui peuvent se présenter chez les personnes âgées^2^. L’IV-IRSC fait la promotion des programmes de recherche et d’autres activités appuyant la recherche qui visent à cerner et à combler les lacunes dans les connaissances, et à identifier les possibilités en matière de promotion de la santé et du bien-être de la population vieillissante du Canada.

L’IV-IRSC considère le parcours de vie dans son ensemble, tout en ciblant spécifiquement les problèmes de santé des individus plus âgés. Il est un leader national dans la détermination des priorités de recherche en santé liées au vieillissement. Les initiatives menées par l’IV-IRSC créent des liens et soutiennent des chercheurs à travers le pays. Elles rassemblent également un vaste ensemble de parties prenantes, dont différents paliers de gouvernement, des praticiens, des organismes de bienfaisance dans le domaine de la santé, des organisations de bénévoles œuvrant dans ce domaine et des personnes âgées. Au début de la crise provoquée par la COVID-19, l’IV-IRSC s’est vite mobilisé pour identifier les domaines de recherche prioritaires liés à cette pandémie et à ses conséquences dramatiques sur les personnes âgées. L’objectif de cet article est de décrire l’approche mise en œuvre par l’IV-IRSC pour saisir rapidement les besoins de la recherche sur la COVID-19 pour les personnes âgées au Canada. Ces priorités permettront d’orienter les prochaines actions de l’IV-IRSC dans le contexte de la COVID-19.

## Actions et consultations

**Figure 1 :**
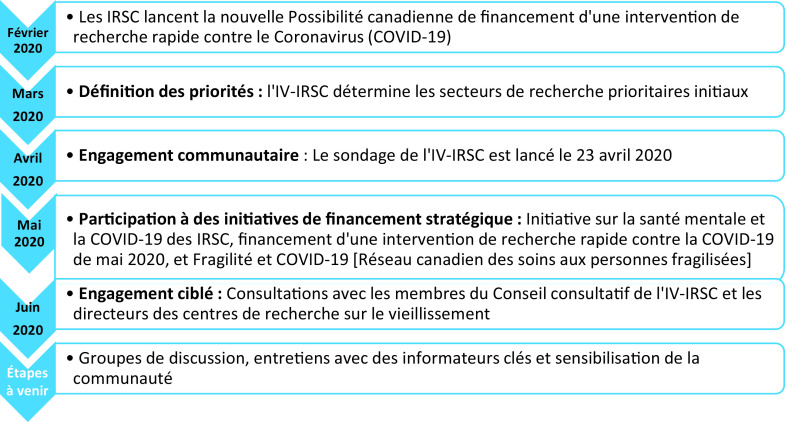
Calendrier des actions pour l’identification des secteurs prioritaires de la recherche concernant l’impact de la pandémie de COVID-19 sur les personnes âgées

Le processus engagé par l’IV-IRSC comprend un certain nombre d’actions et de consultations, tel qu’illustré à la figure 1 et décrit ci-dessous.

Au début du mois de février 2020, les IRSC ont lancé la Possibilité de financement canadienne pour une intervention de recherche rapide contre la COVID-19. Au total, 100 subventions ont été accordées dans le cadre de ce concours impliquant un investissement du gouvernement du Canada de 55,3 millions de dollars, la priorité étant alors la recherche sur les contre-mesures médicales, sociales et politiques. À ce stade précoce de l’identification et de la recherche sur les impacts de la COVID-19 sur la santé, les personnes âgées n’avaient pas encore été reconnues comme une population particulièrement vulnérable. Par conséquent, aucun des projets financés n’était alors axé sur les conséquences de la COVID-19 sur la santé et le bien-être des personnes âgées. Compte tenu de ce résultat et de l’évolution rapide de la situation en lien avec la COVID-19 chez les adultes plus âgés, l’IV-IRSC s’est mobilisé en vue de la définition de priorités de recherche pour répondre aux besoins de la population vieillissante, et se préparer ainsi à participer aux prochaines possibilités de financement proposées par les IRSC et ses partenaires.

Suivant un examen des publications existantes et des données émergentes, l’IV-IRSC a identifié des secteurs de recherche prioritaires initiaux associés à l’impact de la COVID-19 sur les personnes âgées. Un processus accéléré a ensuite été amorcé en vue de faire participer les parties prenantes et mieux comprendre l’impact et les implications de la COVID-19 sur la recherche en vieillissement au Canada. En avril 2020, l’IV-IRSC a diffusé un sondage par ses listes de diffusion à plus de 4 000 de ses abonnés, obtenant ainsi plus de 600 réponses de la part de chercheurs, de stagiaires, de décideurs, d’administrateurs de soins de santé, de fonctionnaires, de fournisseurs de soins de santé, de personnes âgées et de soignants. L’enquête comportait huit questions qui permettaient d’obtenir des réponses quantitatives et qualitatives. Les réponses qualitatives ont été analysées selon une approche thématique et classées en fonction de thèmes émergents. Au début du mois de juin, l’IV-IRSC a entrepris des consultations ciblées avec des parties prenantes clés, incluant les membres du Conseil consultatif de l’Institut du vieillissement et les directeurs des 35 centres de recherche canadiens sur le vieillissement. Les informations issues de ces consultations ont été fusionnées avec les données obtenues lors du sondage, ce qui a permis d’identifier plusieurs secteurs de recherche prioritaires.

## Principales priorités de recherche

**Figure 2 :**
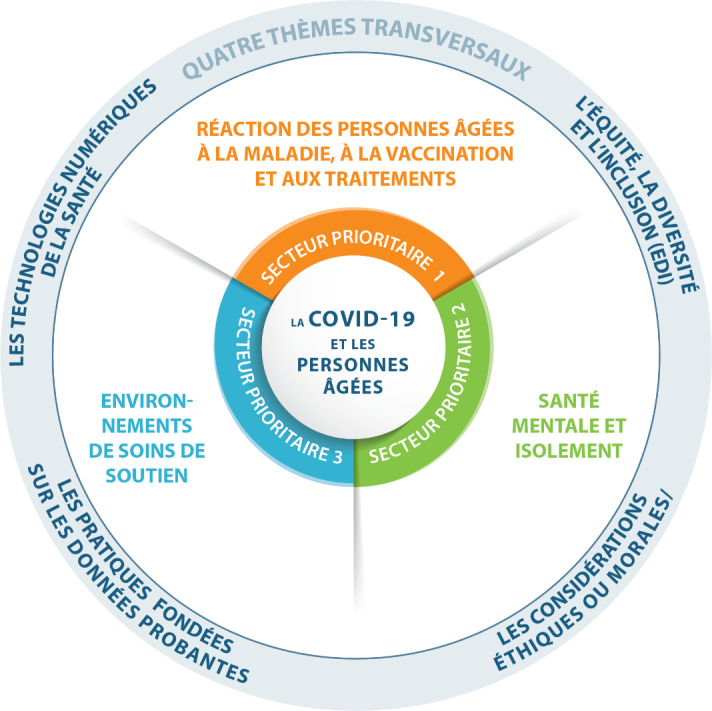
Image infographique illustrant les secteurs prioritaires en recherche et les thèmes transversaux

Le processus d’identification des priorités de l’IV-IRSC a permis de faire ressortir trois secteurs prioritaires et quatre thèmes transversaux visant la recherche sur l’impact de la COVID-19 sur les personnes âgées et le vieillissement, tel qu’illustré à la figure 2.

## Secteur prioritaire 1 : Réponses des personnes âgées à la maladie, à la vaccination et aux traitements

Le vieillissement est un processus multidimensionnel impliquant de nombreux mécanismes cellulaires et moléculaires au sein de différents systèmes d’organes. Ceci inclut des modifications fonctionnelles et structurelles du système immunitaire qui se manifesteraient par une diminution de la capacité à lutter contre les infections et par des inflammations chroniques de faible intensité ("inflammaging") (Franceschi & Campisi, 2014). Les facteurs d’immunosénescence et d’inflammation chronique sont impliqués dans diverses maladies liées au vieillissement et sont corrélés avec une mauvaise réponse à la vaccination (Keenan & Allan, 2018). Il s’agit d’un élément important à considérer dans l’élaboration de stratégies de vaccination efficaces pour les personnes âgées. Les impacts du coronavirus sur les systèmes et organes des personnes âgées, ainsi que leur sensibilité accrue aux séquelles de la COVID-19 ne sont pas documentés. Une approche de type géroscience (Kennedy et coll., 2014) pourrait favoriser l’émergence de connaissances indispensables sur l’interaction entre les processus de vieillissement, le développement de maladies chroniques et l’impact du coronavirus sur les systèmes physiologiques.

Le développement des thérapies destinées à traiter la COVID-19 et les essais cliniques qui sont en cours ne permettraient pas nécessairement de saisir l’impact potentiel du vieillissement. Les personnes âgées peuvent présenter des anomalies sur le plan métabolique et dans l’élimination des médicaments, ainsi qu’une plus grande sensibilité aux médicaments et aux interactions médicamenteuses (Mallet, Spinewine, & Huang, 2007). Il est également possible qu’elles doivent prendre plusieurs médicaments pour des maladies chroniques, ce qui amène des problèmes de polypharmacie lors de l’administration des traitements contre la COVID-19. Ainsi, les personnes âgées constituent une population ayant des besoins particuliers en matière de traitement et devraient être représentées de manière adéquate dans les essais cliniques portant sur des médicaments, des traitements ou des vaccins.

Exemples de besoins de recherche dans le secteur prioritaire 1 :

- Recherches visant à étudier l’impact primaire et secondaire du coronavirus sur les systèmes du corps humain et les comorbidités;
- Recherches visant à comprendre l’effet du coronavirus sur le système immunitaire vieillissant, en vue de contribuer à l’élaboration de stratégies de vaccination. La conception des vaccins et les approches de vaccination devront être adaptées aux personnes âgées afin de garantir l’efficacité, la sécurité, la protection et le vieillissement en santé;
- Essais thérapeutiques qui garantissent l’inclusion des personnes âgées des deux sexes, puisque les hommes et les femmes vieillissent différemment et ne répondent pas toujours de la même manière aux traitements;
- Initiatives favorisant un accès équitable et un dosage approprié des produits thérapeutiques et des vaccins, pour protéger les populations à risque, avec une attention particulière portée aux besoins spécifiques des personnes âgées.

## Secteur prioritaire 2 : Santé mentale et isolement

Les personnes âgées sont affectées par une augmentation du stress et des problèmes de santé mentale en raison de leur vulnérabilité accrue au virus et des exigences strictes d’isolement qui ont été imposées pendant la crise provoquée par le coronavirus (Chu, Donato-Woodger, & Dainton, 2020 ; Steinman, Perry, & Perissinotto, 2020). Les programmes de loisirs et de stimulation dans les résidences et les centres de soins de longue durée ont été temporairement interrompus, ce qui a accentué l’isolement et la solitude des individus plus âgés. En effet, l’isolement pourrait être encore plus marqué pour les personnes âgées vivant seules, puisque leurs aidants ne pouvaient leur rendre visite. Aussi, plusieurs services de soins à domicile et programmes communautaires pour les personnes âgées et leurs aidants, tels que les programmes de jour pour adultes, ont été suspendus, ce qui a engendré une perte de soutien (Flint, Bingham, & Iaboni, 2020). De nombreuses personnes âgées ont été placées dans des situations où leur autonomie dans la prise de décisions a été amoindrie, notamment concernant leur indépendance et les soins qui leur sont destinés. Elles ont ainsi le sentiment d’avoir perdu leur capacité d’autodétermination et leur « valeur » (Flett & Heisel, 2020), c’est-à-dire qu’elles ne se sentent plus appréciées, et se retrouvent dépourvues de leur droit d’être écoutées. Pour les personnes âgées, le fait de pouvoir prendre des décisions ou de participer à la prise de décisions concernant leur propre bien-être est un facteur prédictif de la protection de leur santé mentale et physique ^3^. Cet aspect est crucial dans le cas des décisions concernant les soins et l’accès à des ressources médicales pour les aînés fragiles qui ont contracté la COVID-19 (Hubbard et coll., 2020). L’âgisme – soit l’attitude discriminatoire envers les personnes d’âge avancé – peut avoir contribué aux effets néfastes sur la santé et la longévité des personnes âgées ayant contracté la COVID-19 (Fraser et coll., 2020).

Exemples de besoins de recherche dans le secteur prioritaire 2 :

- Développement et évaluation d’outils et de programmes d’intervention pour réduire l’anxiété et atténuer l’isolement social. Ceux-ci pourraient inclure, entre autres, le développement de solutions de soins de santé numériques accessibles et conviviales pour aider les personnes âgées isolées à rester en contact avec la société. Des programmes ou plateformes éducatifs virtuels pourraient être conçus pour les professionnels et les travailleurs de la santé, en vue de leur fournir des outils d’apprentissage traitant des manières de faire face au stress et de protéger leur propre santé et celle des personnes qu’ils soignent.
- Recherche visant à évaluer l’impact de l’isolement sur les fonctions cognitives et le déclin associé à la démence, avec élaboration et réalisation d’interventions. Cela peut inclure le développement d’outils de télésurveillance de la santé et des fonctions cognitives.
- Collaborations impliquant les responsables de l’élaboration des politiques de soins de santé pour la conception de lignes directrices de pratique clinique qui utilisent les données probantes en matière de prestation de soins aux personnes âgées atteintes de COVID-19 dans des contextes où les services et les ressources de soins de santé sont limités. En particulier, le rationnement éventuel des services de soins de santé offerts aux personnes âgées, qui seraient désavantagées dans la priorisation, peut avoir un impact négatif sur leur santé mentale, celle de leur famille et de leurs soignants.

## Secteur prioritaire 3 : Milieux de soins et de soutien

Les personnes âgées vivent dans divers milieux résidentiels avec des niveaux de soutien variables. La crise sanitaire et sociale résultant de la COVID-19 a mis en lumière des problèmes existant depuis plusieurs années dans le système de prestation de soins pour les personnes âgées. Bien que la pandémie ait affecté les personnes âgées partout au Canada, quel que soit leur milieu de vie, les impacts les plus profonds se sont fait sentir dans les centres de soins de longue durée (SLD)^4^. Au Canada, 80 % des décès dus à la COVID-19 se sont produits dans les centres de soins de longue durée, ce qui représente le plus fort pourcentage au niveau mondial parmi les pays ayant déclaré des cas de COVID-19^5^ (Estabrooks, Flood, & Straus, 2020a; Estabrooks, Straus, et coll., 2020b). Toutefois, il n’y a que près de 7 % des personnes âgées au Canada qui vivent en centres de soins de longue durée^6^, tandis que la plus grande partie de la population plus âgée vit dans la communauté et reçoit des services de soins à domicile ou communautaires, ou de l’aide de membres de leur famille pour fonctionner de façon relativement autonome^7^. La quarantaine et la distanciation sociale ont fortement affecté la capacité de ces systèmes de soutien à fournir les soins et les ressources indispensables aux personnes âgées qui vivent de façon autonome. Plusieurs se sont retrouvées laissées à elles-mêmes, encore plus vulnérables et incapables de satisfaire à leurs besoins fondamentaux. En outre, de nombreuses personnes âgées sont atteintes de problèmes de santé pour lesquels elles nécessitent d’avoir accès à des services et à des traitements qui risquent d’être interrompus ou inaccessibles en temps de pandémie. Cette situation peut être exacerbée par des inégalités retrouvées dans le système médical, engendrant une perte de soins essentiels pour plusieurs personnes âgées.

Exemples de besoins de recherche dans le secteur prioritaire 3 :

- Recherches permettant de définir les meilleures pratiques en matière de soins dans les milieux résidentiels hébergeant des personnes âgées. Ceci comprend l’élaboration et la mise en œuvre de lignes directrices fondées sur des données probantes pour la lutte contre les infections, ainsi que la main-d’œuvre et le recrutement du personnel, et les soins pour les populations vulnérables;
- Détermination des besoins et évaluation des plans de mise en œuvre pour la prestation de soins et de services communautaires aux personnes âgées autonomes ou vivant en établissement, dans la période de reprise post-pandémie;
- Élaboration de programmes et de politiques pour la prestation de soins de santé aux personnes âgées par des processus qui tiennent compte de leurs besoins particuliers et qui favorisent leur inclusion dans la prise de décisions. Ceci comprend la prestation et l’accès aux soins nécessaires qui peuvent être offerts à distance afin de répondre aux besoins de santé physique et mentale.

## Thèmes transversaux et facteurs clés dans les domaines de l’éthique, des services de santé, des politiques publiques et de la santé des populations

Quatre thèmes transversaux importants ont également été identifiés. Ceux-ci peuvent s’appliquer aux trois secteurs prioritaires présentés plus haut. Ces thèmes sont les suivants :

## Équité, diversité et inclusion (EDI)

Les IRSC ont mis en œuvre une série de stratégies et de solutions pour favoriser l’équité, la diversité et l’inclusion dans les écosystèmes de la recherche^8^. En cette période de crise associée à la COVID-19, il est essentiel que les chercheurs qui mènent des travaux sur le vieillissement tiennent compte des populations autochtones et des autres populations sous-représentées, des facteurs socio-économiques et culturels, de l’accès équitable aux ressources du système de santé et de l’engagement des patients, spécialement lorsque ceci concerne les personnes âgées.

## Dilemmes éthiques et moraux associés aux soins de fin de vie dans les situations de pandémie ou d’urgence

Les considérations éthiques et morales dans la recherche sur les personnes âgées sont incontournables dans le cadre de la crise de la COVID-19. Les recherches liées à la maladie, au rationnement des ressources, aux soins palliatifs et à l’isolement sont d’une importance capitale.

## Essais visant à déterminer les meilleures pratiques pour les soins aux personnes âgées ayant la COVID-19, le transfert et l’application des connaissances sur les meilleures pratiques relatives à la préparation pour les situations d’urgence

L’IV-IRSC soutient l’inclusion des personnes âgées dans les essais qui visent à établir les meilleures pratiques dans les soins pour la COVID-19. Historiquement, les personnes âgées ont été exclues des essais ayant un impact sur les soins qui leur sont offerts.

## Technologies de santé numériques soutenant les services de santé et la prestation des soins, y compris la découverte de médicaments et la production de vaccins, ainsi que le suivi et les interventions sociales

L’IV-IRSC est co-leader de l’initiative Innovations en cybersanté. Cette initiative soutient des recherches nationales et internationales exceptionnelles dans le domaine de la santé numérique afin d’apporter des solutions pour le maintien et l’amélioration des conditions de vie des personnes âgées. Les recherches qui rassembleront une diversité de parties prenantes permettront d’améliorer la collaboration sur l’ensemble du continuum de la recherche en santé, allant de la recherche fondamentale à la santé publique.

## Résumé et conclusions

Peu après le début de la pandémie COVID-19, il est devenu évident que les personnes âgées étaient le groupe le plus à risque en matière de symptômes complexes et de mortalité. Afin de faire progresser son mandat visant l’amélioration de la santé et des conditions de vie des personnes âgées, l’IV-IRSC a réagi rapidement, notamment par la consultation de parties prenantes et d’experts, en vue de déterminer les besoins de recherche prioritaires liés aux personnes âgées et à la COVID-19. Les principaux domaines de recherche exposés dans le présent document, comprenant la présentation de la maladie chez les personnes âgées, la vaccination, les traitements et les essais cliniques, la santé mentale et les milieux de soins soutenants, continueront de guider les réponses apportées par l’IV-IRSC dans le long terme.

Parallèlement au processus de consultation et à l’identification des besoins de recherche prioritaires, l’IV-IRSC s’est rapidement mobilisé pour s’associer à trois possibilités de financement majeures des IRSC; la Possibilité de financement pour une intervention de recherche rapide contre la COVID-19 de mai 2020, l’Initiative sur la santé mentale et la COVID-19 et la possibilité de financement initiée par le Réseau canadien des soins aux personnes fragilisées (CFN) pour financer des projets de recherche axés sur l’interaction entre la fragilité et la COVID-19 chez les personnes âgées. Les recherches qui seront menées dans le cadre de ces trois programmes fourniront potentiellement les premières réponses aux besoins prioritaires dans chacun des domaines clés identifiés. L’IV-IRSC poursuivra ses travaux pour identifier de nouvelles possibilités pour le développement de programmes et s’associera aux programmes de recherche qui répondront aux besoins des personnes âgées dans ce domaine.

Les quatre thèmes transversaux identifiés lors du processus de consultation permettent de prendre en compte des éléments essentiels dans la recherche sur l’impact de la COVID-19 chez les personnes âgées. Comme pour tout domaine de la recherche en santé, les considérations liées à l’équité, à la diversité et à l’inclusion des populations sous-représentées doivent être intégrées dans le plan de recherche. Les personnes âgées doivent aussi participer de façon significative à tous les aspects de la recherche. En ce qui concerne la situation spécifique des personnes âgées pendant la pandémie de COVID-19, les considérations liées aux questions de santé mentale et aux dilemmes éthiques et moraux concernant les soins de fin de vie en contexte de pandémie ou de situation d’urgence doivent être soigneusement intégrées dans la recherche. La situation actuelle a fait ressortir la nécessité de repenser le statut et le rôle des personnes âgées dans notre société et celle d’examiner spécifiquement l’impact et l’influence de l’âgisme dans la prise de décision et la prestation des soins. La recherche axée sur les politiques publiques visant à réduire l’exclusion sociale des personnes âgées et à lutter contre l’âgisme et la stigmatisation associés à la fin de la vie, à la fragilité et à la vulnérabilité est plus nécessaire que jamais. Il est devenu urgent d’inclure des personnes âgées dans les essais visant à déterminer les meilleures pratiques en matière de soins pour la COVID-19. À l’heure actuelle, avec l’évolution des outils et des techniques, la recherche en santé numérique doit être utilisée pour apporter des solutions permettant de préserver et d’améliorer la vie des personnes âgées.

L’IV-IRSC vise à assurer que les besoins en recherche dans le domaine du vieillissement au Canada soient comblés. Son engagement se traduit par l’appui aux programmes de recherche axés sur le développement d’approches permettant de contribuer à la santé et au bien-être des personnes âgées pendant la pandémie de COVID-19 et au cours de la période de reprise post-pandémique.

## Notes

1
https://www.who.int/dg/speeches/detail/who-director-general-s-opening-remarks-at-the-media-briefing-on-covid-19---11-march-2020

2 Institut du vieillissement des IRSC, Plan stratégique 2019–2021 : Vivre vieux, vieillir mieux, https://cihr-irsc.gc.ca/f/46837.html

3
https://news.yorku.ca/2020/06/16/feeling-expendable-during-the-pandemic-why-older-adults-need-to-feel-like-they-matter/

4
https://www.canada.ca/fr/sante-canada/services/soins-domicile-et-soins-continus/soins-offerts-etablissements-soins-longue-duree.html

5
https://www.cihi.ca/fr/les-soins-de-longue-duree-et-la-covid-19-comparaisons-internationales

6
https://www.statcan.gc.ca/fra/sujets-debut/aines_et_vieillissement

7
https://www.canada.ca/fr/sante-canada/services/soins-domicile-et-soins-continus/soins-domicile-et-soins-communautaires.html

8
https://cihr-irsc.gc.ca/f/50068.html

## References

[r1] Chu, C. H., Donato-Woodger, S., & Dainton, C. J. (2020). Competing crises: COVID-19 countermeasures and social isolation among older adults in long-term care. Journal of Advanced Nursing, 76(10), 2456–2459. doi.org/10.1111/jan.14467. PMC7361866 32643787

[r2] Estabrooks, C., Flood, C., & Straus, S. (2020a). We must act now to prevent a second wave of long-term care deaths. *The Globe and Mail* Repéré à https://www.theglobeandmail.com/opinion/article-we-must-act-now-to-prevent-a-second-wave-of-long-term-care-deaths/.

[r3] Estabrooks, C. A., Straus, S., Flood, C. M., Keefe, J., Armstrong, P., Donner, G., … Wolfson, M. (2020b). Rétablir la confiance : la COVID-19 et l’avenir des soins de longue durée. Une note de breffage du groupe de travail sur les soins de longue durée. Société Royale du Canada. Repéré à https://rsc-src.ca/sites/default/files/LTC%20PB%20%2B%20ES_FR_0.pdf.

[r4] Flett, G. L., & Heisel, M. J. (2020). Aging and feeling valued versus expendable during the COVID-19 pandemic and beyond: A review and commentary of why mattering is fundamental to the health and well-being of older adults. International Journal of Mental Health & Addiction. doi.org/10.1007/s11469-020-00339-4. PMC7295320 32837430

[r5] Flint, A. J., Bingham, K. S., & Iaboni, A. (2020). Effect of COVID-19 on the mental health care of older people in Canada. International Psychogeriatrics, 1–4. doi.org/10.1017/S1041610220000708. PMC7235298 32326993

[r6] Franceschi, C., & Campisi, J. (2014). Chronic inflammation (inflammaging) and its potential contribution to age-associated diseases. The Journals of Gerontology: Series A, 69(1), S4–S9. doi.org/10.1093/gerona/glu057. 24833586

[r7] Fraser, S., Legace, M., Bongue, B., Ndeye, N., Guyot, J., Bechard, L., … Tougas, F. (2020). Ageism and COVID-19: What does our society’s response say about us? American Psychologist. doi.org/10.1037/amp0000699. PMC7239227 32377666

[r11] Hubbard, R. E., Maier, A. B., Hilmer, S. N., Naganathan, V., Etherton-Beer, C., & Rockwood, K. (2020). Frailty in the face of COVID-19. Age and Ageing, 49(4), 499–500. doi.org/10.1093/ageing/afaa095. 32374368 PMC7239250

[r14] Instituts de recherche en santé du Canada. (2019). Priorités de recherche stratégique de l’IV 2019–2021. Repéré à https://cihr-irsc.gc.ca/f/46837.html.

[r15] Keenan, C. R., & Allan, R. S. (2018). Epigenomic drivers of immune dysfunction in aging. Aging Cell, 18(1), 1–15. doi.org/10.1111/acel.12878. PMC6351880 30488545

[r16] Kennedy, B. K., Berger, S. L., Brunet, A., Campisi, J., Cuervo, A. M., Epel, E. S., … Sierra, F. (2014). Geroscience: Linking aging to chronic disease. Cell, 159, 709–713. doi.org/10.1016/j.cell.2014.10.039. 25417146 PMC4852871

[r17] Mallet, L., Spinewine, A., & Huang, A. (2007). The challenge of managing drug interactions in elderly people. Lancet, 370(9582), 185 Repéré à 10.1016/S0140-6736(07)61092-7. 17630042

[r20] Steinman, M. A., Perry, L., & Perissinotto, C. M. (2020). Meeting the care needs of older adults isolated at home during the COVID-19 pandemic. JAMA Internal Medicine, 180(6), 819–820. 10.1001/jamainternmed.2020.1661. 32297903

